# Positive deviance control-case life history: a method to develop grounded hypotheses about successful long-term avoidance of infection

**DOI:** 10.1186/1471-2458-8-94

**Published:** 2008-03-20

**Authors:** Samuel R Friedman, Pedro Mateu-Gelabert, Milagros Sandoval, Holly Hagan, Don C Des Jarlais

**Affiliations:** 1 National Development and Research Institutes, Inc. 71 West 23d Street, 8^th ^floor, New York, NY 10010 USA; 2 Department of Epidemiology, Bloomberg School of Public Health, Johns Hopkins University, Baltimore, MD, USA; 3 Baron Edmond de Rothschild Chemical Dependency Institute, Beth Israel Medical Center, New York, NY, USA

## Abstract

**Background:**

Prevalence rates for long-term injection drug users in some localities surpass 60% for HIV and 80% for HCV. We describe methods for developing grounded hypotheses about how some injectors avoid infection with either virus.

**Methods:**

Subjects: 25 drug injectors who have injected drugs 8 – 15 years in New York City. 17 remain without antibody to either HIV or HCV; 3 are double-positives; and 5 are positive for HCV but not HIV. "Staying Safe" methodology compares serostatus groups using detailed biographical timelines and narratives; and information about how subjects maintain access to physical resources and social support; their strategies and tactics to remain safe; how they handle problems of addiction and demands by drug dealers and other drug users; and how their behaviors and strategies do or do not become socially-embedded practices. Grounded theory and life-history analysis techniques compare and contrast doubly-uninfected with those infected with both viruses or only with HCV.

**Results:**

Themes and initial hypotheses emerging from analyses included two master hypotheses that, if confirmed, should help shape preventive interventions: 1) Staying uninfected is not simply a question of social structure or social position. It involves agency by drug injectors, including sustained hard work and adaptation to changing circumstances. 2) Multiple intentionalities contribute to remaining uninfected. These conscious goals include balancing one's need for drugs and one's income; developing ways to avoid drug withdrawal sickness; avoiding situations where other drug users importune you to share drugs; and avoiding HIV (and perhaps HCV) infection. Thus, focusing on a single goal in prevention might be sub-optimal.

Other hypotheses specify mechanisms of enacting these intentionalities. One example is finding ways to avoid extreme social ostracism.

**Conclusion:**

We have identified strategies and tactics that some doubly-uninfected IDUs have developed to stay safe. Staying Safe methodology develops grounded hypotheses. These can be tested through cohort studies of incidence and prevention trials of hypothesis-based programs to help drug injectors make their injection and sexual careers safer for themselves and others. This positive deviance control-case life history method might be used to study avoiding other infections like genital herpes among sex workers.

## Background

Highly-infectious endemic sexually-transmitted or parenterally-transmitted diseases typically infect large percentages of susceptibles within a few years of their beginning to have sex or to inject drugs. This is true of hepatitis C for illicit drug injectors [1], human papillomavirus for sexually active people [2], and herpes simplex, Type 2, for commercial sex workers [3].

Epidemiology has developed many methods to determine the risk factors for becoming infected. For hepatitis C among drug injectors, for example, these include the number of years a person has injected drugs, receptive syringe sharing, and sharing the "cookers" in which drug mixtures are prepared and from which the solution is extracted by users' syringes [4-12]

For these highly-infectious diseases, however, knowing the risk factors may not be adequate to suggest effective prevention methods. For endemic hepatitis C, for example, the combination of the infectivity of the virus, the high prevalence of infectious people in drug injector populations, and the socially- or personally-created difficulties of avoiding all risk combine to render prevention only marginally effective. Thus, in spite of a large and well-developed set of programs to prevent the spread of HIV among drug injectors, among people who had been injecting drugs between eight and fifteen years, approximately 70% in both Sydney, Australia, and London, England, were infected by hepatitis C [13]. Most researchers and public health workers in the field believe that there is a dire need for new approaches to prevention. In the absence of an effective preventive vaccine, or perhaps even after one is developed if it proves as difficult to arrange vaccination of injectors against hepatitis C as it has proved to be for hepatitis B [14,15], such prevention will have to be sociobehavioral.

One approach might be to ask how the long-term injectors who have remained uninfected with hepatitis C have done so. Current research methods, however, do not provide a way to study how these uninfected injectors (or parallel uninfected members of other populations, such as long-term sex workers who have avoided genital herpes) have managed to stay safe. (See the beginning of the Methods section for discussion of how potential differences in biological susceptibility need to be taken into account.)

**Figure 1:**
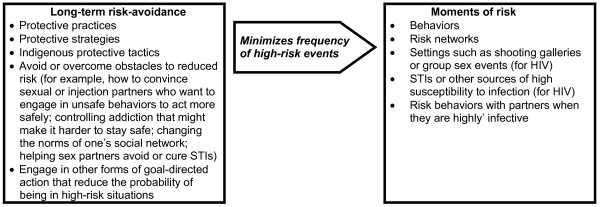
Hypothesized relationship between long-term risk avoidance and short-term risk for HIV or HCV infection

Determining how some people stay uninfected over the long term is not just a question of reversing the signs in a risk factor equation. This is because what we need to learn is how and why the uninfected people were able to avoid high-risk situations and behaviors sufficiently well so as not to have become infected (See Figure 1).

Thus, a risk factor study focuses on what behaviors, partners, personal biological factors, and environmental factors come together to lead to one or more short-term events in which a virus is transmitted to and infects a subject. To understand long-term non-infection, however, we must study what patterns of long-term behavior and social interaction, and thus what strategies and practices of risk-avoidance, differentiate IDUs who are infected from those who are not infected.

Even the terminology we need to address this issue may seem unusual to some readers. Strategies are purposeful and planned patterns of behaviors that are meant to attain one or more goals. This can get complicated. Strategies to prevent infection are one thing; but other strategies, such as strategies to maintain some control over one's addiction, may be equally or more important in long-term prevention of HIV and HCV. Practices are socially-embedded and socially-meaningful behaviors or sets of behaviors. Although momentary acts or behaviors can lead to getting infected (or infecting someone else), socially-embedded and socially-meaningful patterns of behavior (such as trying to maintain respectability in the eyes either of non-user relatives or of "righteous dope fiends") involve long-term maintenance of safer (or riskier) behaviors.

We present here a methodology through which to discover grounded hypotheses [16,17]. by investigating differences between those who stay uninfected over the long haul and those who become infected. This methodology can, with some margin of error, uncover and develop hypotheses about what circumstances, practices and strategies protect people from infection by comparing outwardly-high-risk people who are not infected with those who are infected–with the emphasis being to study how the uninfected controls differ from the infected cases. This requires that we study people who have embodied these practices and strategies for a long enough time for the oddities of chance (as expressed in the probabilistic nature of viral transmission and of having a partner who is infectious) to even out.

### Positive Deviance Research

Researchers in child nutrition in developing countries have found that, even in communities where malnutrition is common, some children are not malnourished [18,19] Using the concept of "positive deviance," they study the practices and other characteristics of the few families whose children are well-nourished even though their access to resources was similar to that of other families. In the words of a summary document about this approach, these "characteristics may be behavioral, social, psychological, or physiological [20]." The method is generally applied to high-risk circumstances–like communities with high rates of malnourished children, or IDU communities where HCV and HIV are highly prevalent–and "focuses on identifying sources and pathways of natural immunity or adaptive resistance." The summary document [20] adds, "positive-deviance studies tend to require complicated designs and analyses, because many psychological and behavioral factors contribute to resistance." We would add that many social and environmental factors must also be considered. The concept of positive deviance seems to overlap with other concepts such as resilience, hardiness, well-being, and wellness, but may differ inasmuch as it focuses relatively more attention on the practices and strategies that succeed in producing positive outcomes rather than upon the individual personality traits that contribute to resilience. Resilience, for example, is more about personality traits and the effects of external social factors such as social support and involvement in activities [21-23]) whereas Positive Deviance is more about strategies and practices that are protective.

Positive deviance approaches in HIV research have focused on studying individual-level factors associated with the absence of risk behaviors. Save the Children Federation conducted focus groups among Vietnamese IDUs and commercial sex workers to elicit specific ways in which they try to minimize their risks [24]. For IDUs, the specific practices identified were (1) using a syringe only if it was sealed in the package; (2) bending the needle after use to prevent re-use; (3) telling those who asked that they could not share needles because it could make them ill; (4) getting syringes at a reduced rate at a pharmacy; and (5) sniffing drugs rather than injecting if no clean syringe was available. For sex workers, approaches identified were (1) successfully negotiating condom use by telling clients that she was concerned about their family getting ill; (2) if no condom was readily available, telling the customer that she wanted to put on something more attractive for him–while asking a co-worker to go to the pharmacy and buy condoms; and (3) asking if there was a particular type of condom that a customer would like to use, and, if this was not available, stating that they should use what was available, but next time they would have their preferred brand.

Babalola studied current lower risk behavior among Rwandan youth as positive deviance [25]. Factors related to sexual abstinence include non-urban residence, younger age, Christian religion, not using alcohol, perceptions about peers' sexual behaviors, self-esteem, perceived self-efficacy to refuse sex with someone known for more than 3 months, perceived self-efficacy to refuse sex with someone truly loved and attitudes toward premarital sex. Thus in this study as in a study of West African youth [26], they focus on beliefs, values, and support in the recent past.

These studies focus on identifying safer behaviors or tactics to negotiate safer behaviors and/or the relatively immediate factors associated with safer behaviors. This is, of course, extremely useful, and is quite parallel to innumerable studies of risk and non-risk in the HIV and HCV fields. It is important to understand the difference between these studies of positive deviance and those which we are discussing in this paper. As opposed to their primary emphasis on immediate or recent behaviors, our methodology studies how some IDUs remain uninfected over many years of injection drug use. Thus, our emphasis is in terms of long-term trajectories in strategies, conditions, practices, and events that shape sustained, long-term safer behavior and/or long-term safer networks. This requires more detailed data covering long time periods, including issues such as how they react when friends or family go to or return from prison, get sick, or die. Research methodologies such as the focus groups used by Save the Children Federation in Vietnam are a good way to elicit tactics. To discover and understand strategies, however, requires the more in-depth life history methodologies we use.

## Methods

The essential methodology is to recruit a group of controls who have seemingly been at risk for a long period without becoming infected and to compare it to an appropriate comparison group of cases who have been infected. An important component of this research is thus to find study sites and subjects for whom having remained uninfected is indeed an accomplishment; in practice, this means looking at data on infection markers by years of potential exposure.

Since the focus of this method is to explore ways in which those at risk run their lives so as to remain uninfected, it is important to minimize the probability that the negative controls have remained uninfected for biological reasons such as having immunity of a kind that does not show up on standard assays. One way to do this is to focus on more than one infection at a time. Thus, in New York we are studying how IDUs remain uninfected with both HIV and HCV by comparing these IDUs to those who are infected with both and those who are infected only with HCV. (The proportion that is infected only with HIV is quite small, and is more likely to reflect biological causation.) Although the fact that HIV prevalence among IDUs at this time is "only" 19% [27] reduces the value of HIV negativity as a way to rule out low biological susceptibility to hepatitis C seroconversion, Aitken et al., (2004) [28] have found a very low probability of falsely classifying subjects' hepatitis C status due to clearing prior infection (but remaining antibody-positive) or natural immunity. Nonetheless, there may be a small proportion of injection drug users who become exposed to hepatitis C but clear the virus without becoming antibody positive [29,30], and this does pose some risk of misclassification–and supports the importance of focusing on more than one infection where possible.

Figure 1 provides an overview of the processes that this method addresses. Long-term risk avoidance involves minimizing being in situations and engaging in behaviors that pose a high risk of infection, and, when one is in a high-risk situation, having a repertoire of methods to avoid risk anyway. This means that the research focus is on practices that are socially-embedded in one's social network, as well as on how a person or small group of people avoid or resist social pressures to take risk. We want to learn from the experts–those who have avoided infection–what they have done to stay safe, what they have done for other reasons that has also helped them to stay safe, how they learned to act in these ways, and how they managed to maintain relatively safe behavior over a period of many years while engaging in behaviors (drug injection, selling sex) that, on the surface, seem to pose a high risk of leading to infection.

### Recruiting the participants and eligibility issues

The logic of the project requires that participants will have been at risk (in this case, injecting drugs) long enough for most of their cohort to have become infected with one or both of the infectious agents being studied. A second component to this logic may be less obvious–the value of an upper bound on the exposure time for eligibility. This upper bound serves two primary substantive functions. The first is that we are generally going to try to understand how people stay safe under more or less current conditions, so we want to restrict the time period to a period in which conditions have been more or less similar. For New York, syringe exchange on a large scale began about 15 years before the project began gathering data, and this greatly reduced the difficulties of obtaining sterile injection equipment. The second reason an upper bound is useful is that it may restrict the effects of mortality upon the sample of infected participants. People who are infected with HIV (particularly if they are also infected with hepatitis C) had a very high mortality rate before the late 1990s–but this mainly became true more than five years after they became infected. Thus, for IDUs in New York, our criterion of recruiting IDUs with 8 – 15 years of injection experience (with interviews beginning in 2005) meant that they had all begun injecting after syringe exchange opened; had mainly injected after the subsequent great decline in incidence rates [31], and would have been very unlikely to have become infected early enough to have died before HAART became widely used. Finally, these criteria would recruit a sample of double-negatives who were positive deviants since only 25% of New York City IDUs in a reasonably representative detox treatment entry sample who had been injecting for 8 – 15 years remained both HIV and HCV negative [13].

Similar patterns of reasoning motivated colleagues in Australia and in England to propose 8 – 15 years of injecting as their sampling frame for studies of how IDUs there had remained uninfected with hepatitis C [13].

Since we are deliberately seeking out positive deviants (a relatively rare group) as half of our overall sample, there is considerable cost saving in studying subjects who are referred to us by research or other sites who themselves do the appropriate testing for HIV and hepatitis C antibody and also screen subjects by how long they have been injecting drugs. On the other hand, this has led to slow recruitment when these sites have other priorities, and also limits our sample to those who use the sites.

Two sources recruited the subjects included in this paper: First, the Risk Factors project at Beth Israel Medical Center detoxification center, which has been the subject of many research papers [27,31] was the referral source for five subjects who were negative for both viruses, one who was positive for both viruses, and two who were positive only for hepatitis C. No one they approached to take part in the Staying Safe Project told them that they would not participate or refused to provide them with information about how we could reach them. However, since some time elapsed before antibody test results became available, it was not always possible to reach potential subjects to schedule an interview. This may have resulted in our sample under-recruiting IDUs with unstable lives. HIV testing was conducted at the New York City Department of Health Laboratory using repeated enzyme-linked immunosorbent assays (ELISA) testing with Western blot confirmation. Hepatitis C testing there used Abbott HCV EIA 2.0 (Hepatitis C Virus encoded Antigen (Recombinant c100-3, HC-31, andHC-34); confirmatory testing (RIBA HCV 3.0 SIA) was performed when absorbance values were equal to or less than 3.8: Chiron RIBA HCV 3.0 SIA (Hepatitis C Virus encoded antigen (Recombinant c33c and NS5 antigens;Synthetic 5-1-1, c100 and c22 peptides).

Second, the Etiology of Bloodborne Viral Iinfections project used respondent-driven sampling to recruit a relatively representative sample of injectors, and referred those meeting our criteria to us for interview. All eligible subjects whom they referred were interviewed. It was the referral source for twelve subjects who were negative for both viruses, two who were positive for both viruses, and three who were positive only for hepatitis C. Their sera were screened for anti-HCV with a third generation enzyme immunoassay (Abbott Laboratories, Chicago, Illinois); and anti-HIV testing was performed using licensed ELISA screening and western blot confirmatory tests.

### Eliciting interview data

To elicit such data, we engage in a combination of questions that help us understand their overall biography and the threats and resources this has involved; their history of risk and risk avoidance; and detailed elicitation of some of the key concepts presented in Figure 1.

#### Eliciting biographical information

**Figure 2:**
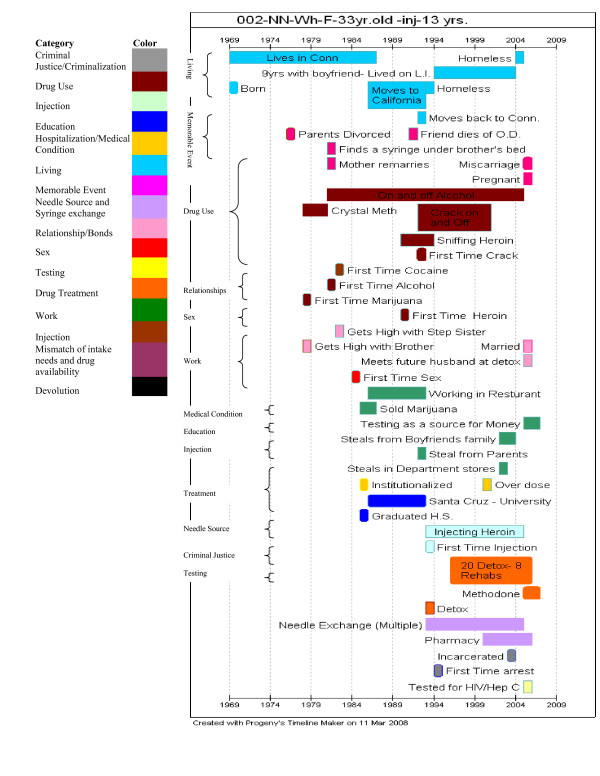
**Example of a Time Line for a Participant**. The participant is a white 33 year old woman who is HIV-negative and HCV-negative. She has been injecting 13 years.

Life histories are elicited in a two-step process. First, we work with the participant to draw a visual timeline to serve us as a schematic reminder of the participant's overall life and, equally or more important, to serve as an easily-accessible reference during the remainder of the interview. (We enter this into TimeLine software after the interview since doing it during the interview is cumbersome and disrupts the natural flow of a person's describing her or his life.) An example of such a timeline appears as Figure 2.

These timeline diagrams provide a valuable resource in analysis that helps us identify patterns in the life history data such as the relatively large number of years it took some subjects between starting to inject and beginning to use needle exchange.

After this, we interview the subject with a more detailed biographically-structured interview guide (see Additional file 1) that elicits narrative about their history and experiences with drug use, access to drugs, sexual experiences and relationships, institutional experience (including medical history, hospitalization, incarceration and similar issues), knowledge and practices of HIV and hepatitis C and related issues, and strategies and tactics to avoid stigma, high risk situations, and infection.

#### Eliciting additional information about protective tactics, strategies and environments

During this interview, and particularly at the end of it, as well as in follow-up interviews, we try to understand the social processes that lead to these tactics, strategies and behaviors. Issues here include:

1. *Practices versus behaviors: *Probes about the extent to which particular behaviors or behavioral patterns are socially-embedded practices or are more individualized behaviors. This is not easy interviewing, because there is a strong tendency in the drug culture as well as in the society as a whole [32] to describe one's behaviors as expressions of one's personality and choices. One approach we take to probing this is to ask them about how they learned to do things that way–since one perspective on culture is that it consists largely of learned behavior. Another approach is to ask them about how others would react if they behaved in other ways, which gets at the processes by which norms are maintained [33-37]

2. *Indigenous prevention tactics*: One of our goals is to identify indigenous prevention tactics–that is, behavioral rules of thumb or practices which help subjects control their risk that have immediate face validity as possible prevention messages. These differ from trajectories, resources, and strategies in that they are immediate and do not necessarily address the issue of long-term maintenance of safer practices (including maintaining indigenous prevention tactics).

3. *Strategic action: *To determine whether a subject's particular pattern of action is strategic (i.e., planned and intentional), we probe about why they engaged in the pattern of action; the extent to which they planned it out; what they had in mind; whether it emerged out of group discussions, and if so, what the group was trying to accomplish if anything. Given the frequently-changing situations IDUs find themselves in, particularly in those periods of their lives when they are street users, they sometimes have trouble recognizing the extent to which their actions indeed are based on planning and intentionality rather than being overwhelmingly circumstantial and reactive. We have uncovered some of their strategies in these circumstances by asking them how they apply their principles of infection avoidance, for example, to different circumstances they face; and asking them to comment of how and why they sometimes act differently in various circumstances from how those with them act.

#### Personality data about traits

One possible explanation for why some IDUs might be able to remain uninfected would be if they share some common personality trait or traits that remain reasonably constant over the many years they have been injecting drugs. To test for this, we use selected parts of the NEO PI-R questionnaire. This questionnaire is based on Costa & McCrae's Five Factor Model of Personality (FFM), which is a highly influential theory of personality. It provides a relatively comprehensive taxonomy of individual differences. The five domains are *Neuroticism*, the tendency to experience negative affect (e.g., anxiety, depression, and hostility); *Extraversion*, the quantity and intensity of interpersonal interactions; *Openness to Experience*, seeking and appreciation of new experiences; *Agreeableness*, the quality of interpersonal interactions along a continuum from compassion to antagonism; and *Conscientiousness*, the amount of persistence, organization, and motivation in goal-directed behaviors [38,39]. These factors show extraordinary stability over time in normal populations, with little change over decades [39,40] (The NEO PI-R contains 240 items. It can be completed in 30 – 40 minutes).

#### Interview Contexts

Interviews were conducted under confidentiality and other protocols approved by the institutional review board of NDRI at a storefront field site located within a few blocks of one referral site; another source referred participants to us from several locations (including one in which they rented space in a separate part of our storefront). The different sections of the interviews took, on average: 55 minutes for the Time Line; 81 minutes for the detailed interview about protective strategies and related matters in the context of their life history; and, in seven cases when follow-up interviews have been conducted to elicit more information about these topics, with special attention to strategies and to the sharing of equipment other than syringes, approximately another 80 minutes. Participants are reimbursed $40 for their time and trouble for the first interview and $30 for any subsequent interviews.

#### Analyzing the data to develop grounded hypotheses

Data analysis follows fairly standard techniques from grounded theory and life history methodologies. All interviews are taped and transcribed. The transcripts are then coded by both of the field staff using a combination of theory-based and emergent coding categories [17], who also discussed and resolved differences in coding. The Principal Investigator read selected transcripts as well. Discussions during project meetings helped new concepts to emerge; there were often concepts that were more abstract or process-oriented than those that emerged during the coding itself. As staff developed ideas about important processes, categories, or concepts, they wrote and circulated theory notes for further discussion by project members.

Due to difficulty recruiting infected subjects during the early months of the project, the first set of emergent codes were dominated by the materials from subjects who had remained uninfected–that is, from those who had successfully "stayed safe." Later, as we interviewed IDUs who had been infected with HCV and in some cases also with HIV, categories began to emerge that reflect their lives and, usefully, the comparisons between the positives and the negatives. Additional analyses may be conducted comparing those who are infected with both HIV and HCV with those who are infected with HCV but not HIV.

These categories and concepts are partially reflected in the hypotheses presented in the Results section below. Since both data collection and analysis are ongoing in this project, we anticipate that additional grounded hypotheses will be developed and those presented here may be modified.

## Results

Since this paper is primarily methodological, we simply list here some of the hypotheses we have developed based on interviews so far with 25 IDUs, of whom 17 were doubly-uninfected, 3 doubly-infected, and 5 infected with HCV but not with HIV. Several themes and initial hypotheses have emerged from the data.

First, we propose two master hypotheses that, if confirmed, should be taken into account in forming preventive interventions:

1. Staying uninfected is not simply a question of social structure or social position. It involves agency on the part of IDUs as well. Thus, it is not simply well-to-do IDUs who can avoid risk. Many IDUs whom we interviewed remained uninfected with either HIV or HCV in spite of five years or more of homelessness, arrests, exposure to violence, and serious poverty; though a few of our uninfected IDUs had started out relatively well off and still remain so. Thus, to a large extent, IDUs have agency, and remaining uninfected is a result of considerable sustained hard work and adaptation to changing circumstances.

2. Multiple "intentionalities" are hypothesized to help contribute to remaining uninfected. Developing successful ways to maintain a balance between one's need for drugs and one's income, and in other ways to insure access to drugs, is one dimension of this. Another is developing ways to stave off drug withdrawal sickness which is an extremely unpleasant experience that drug users will do much to avoid (and that also, as a result, poses a heightened probability of engaging in behaviors that risk infection). A third is trying to avoid situations in which other drug users will importune you to share drugs. A fourth is trying to avoid infection with HIV. A fifth, which in many but not all localities is secondary to HIV-avoidance [41] is avoiding infection with hepatitis C. An important implication of this hypothesis should be noted: This hypothesis means that focusing on a single goal in prevention might be a sub-optimal approach. Individual-level focused interventions that use cognitive-behavioral programs like the AIDS Risk Reduction Model [42], Health Belief Model [43], Information-Motivation-Behavioral Skills [44], Social Cognitive Theory [45,46], Social Action Theory [47], Theory of Planned Behavior [48,49], and Transtheoretical model [50-52] may be less effective than programs that address more than one, or all, of the above dimensions of intentionality.

In addition, we hypothesize that the following more specific practices, strategies and other characteristics of IDUs form part of a complex and changing (as their lives change) set of adaptive repertoires and other responses and conditions that help keep IDUs uninfected:

1. Developing ways to avoid "drug sickness" (withdrawal) by keeping drug need commensurate with money and drugs availability such as maintaining close relationships with drug dealers so they will lend you drugs; being a mid-level dealer oneself; or using detoxification programs to manage one's "habit."

2. Developing ways to ameliorate or to cope with drug sickness episodes without engaging in high-risk injecting. These methods seem to include planning ahead or developing social partnerships so you get sterile syringes, whether from syringe exchanges, pharmacies, or diabetics (both when in the community and when incarcerated); sniffing drugs rather than injecting them when caught without a sterile syringe; and going cold turkey without drugs until they can get a clean syringe and finding ways to distract themselves from their discomfort.

3. Developing ways to reduce peer pressure to share drugs or equipment such as teaching lovers and friends to save "wake-up bags"; injecting alone or with a like-minded partner; and teaching and reinforcing safety practices in one's own injection network.

4. Developing ways to handle stigma and to avoid "social death." These include avoiding being identified by family, friends or neighbors as an injector; using harm reduction programs as sources of social support and friendship; and never violating the space or property of some friends and relatives so they will remain resources as a place to keep papers, shower and/or sleep occasionally.

## Discussion and Conclusion

We have identified strategies, tactics and practices that we hypothesize have helped some IDUs remain both HIV-negative and HCV-negative over 8 to 15 year long injection careers. Clearly, as indicated by Figure 1, these strategies, tactics and practices take place in a probabilistic environment. Thus, some IDUs have remained doubly-uninfected even though they sometimes share syringes. Others seem to have become infected with hepatitis C from single instances of high-risk injection. This is suggested by two subjects for whom we have two sets of test results a few months apart; in each case, interviewing them about what happened identified specific instances of syringe sharing that we quite unusual and contra-normative for them.

They also take place in an environment in which one potential structural limitation on the ability of IDUs has been greatly reduced. This is syringe availability. Unlike the circumstances during the early years of the epidemic in New York, sterile syringes are now legally accessible and comparatively plentiful.

Some (but not all) of these staying safe techniques, such as controlling withdrawal and maintaining good sources of drugs, were previously pointed to as strategies that had been engaged in by long-time injectors who had survived into their 50's [53]. Although these findings remain tentative, and may vary to some extent depending on the drugs being used and variations in drug markets, we do think they point the way to potentially-improved prevention approaches and perhaps even a new generation of programs to help IDUs make their injection and sexual careers safer for themselves and others. Since researchers in Spain, Australia, the Czech Republic and England are adapting Staying Safe methodology to study long-term HCV and/or HIV avoidance; collaboration among us will widen our ability to learn about and disseminate successful innovations and to understand the implications of different patterns of drug use (such as the proportions of injectors who mainly inject heroin versus atimulants) on the hypothoses that are developed.

### Methodologically

Staying Safe methodology helps develop grounded hypotheses about how outwardly-high-risk people remain uninfected.

### Hypothesis-testing

Such hypotheses of course need to be verified epidemiologically through cohort studies of incidence and/or through prevention trials that test these hypotheses together with testing ways to apply them in practice.

The positive deviance control-case life history method should be applicable to studying ways in which people remain safe from other infections like genital herpes among sex workers. Indeed, we suspect it may provide a mechanism to discover new ways to prevent a variety of highly-prevalent infectious diseases, and perhaps behavioral disorders that have proven resistant to existent techniques.

## Abbreviations

HCV Hepatitis C Virus

HIV Human Immunodeficiency Virus

IDU Injection drug user

## Competing interests

The author(s) declare that they have no competing interests.

## Authors' contributions

SRF conceived the study and led in designing it. He also conceived of and drafted this paper, as well as taking part in the analysis of the data.

PM-G helped design the study, led and participated in all aspects of the field work, took part in the data analysis, and helped to write the manuscript.

MS helped design the study, led and participated in all aspects of the field work, took part in the data analysis, and helped to write the manuscript.

HH helped design the study and took part in writing the manuscript.

DCD helped design the study and took part in writing the manuscript.

All authors read and approved the final manuscript.

## Pre-publication history

The pre-publication history for this paper can be accessed here:



## Supplementary Material

Additional File 1 Appendix 1. Life History Protocol. This is the interview guide for eliciting life history information. Click here for file
