# Supplementary material for: Positive deviance control-case life history: a method to develop grounded hypotheses about successful long-term avoidance of infection
Source: BMC Public Health. 2008 Mar 20;8:94. doi: 10.1186/1471-2458-8-94 (PMC2329618; doi:10.1186/1471-2458-8-94)
Supplement: Additional File 1 — Appendix 1. Life History Protocol. This is the interview guide for eliciting life history information. [file 1471-2458-8-94-S1.doc]

# Appendix 1. Life History Protocol

*Note to interviewer: This life history protocol is designed to adopt and adapt to the main life periods identified by the subject during the timeline. Those periods are likely to follow a chronological order within those time periods described by the subjects.*

*If the respondent has difficulty identifying his/her own time periods use the ones indicated in brackets as alternative. In general, these periods will be marked by important changes in subjects ‘physical & social environments. The transitions and changes should be recorded during the interview.*

*Some examples of subject’s driven periods:*

- *Before my first child (0-16); Happy days-Manageable drug use(16-20); Heavy drug use (20-25), clean 25-30)*
- *Days in Puerto Rico (0-10), foster care-NY 12-16), Money days-drug dealing (18-23), tough days-incarceration (23-30), Out (30-31)*
- *Childhhood-Army- Drugs- Incarceration-*

Tell me a bit about yourself….

## Period I [Childhood, 0-12 years]

Where were you born?

How was your family growing up? (Mom, Dad, Brother’s, Sister’s) Religion

How was the neighborhood growing up?

What about School, did you go to school, did you like school?

### Period II [Early Teens, 12-16 years old]

What were the major differences between this time period and the previous time period?

Relationship with parents/family?

So what was going on with school?

What did you like to do when not dealing with school? Any hobbies or interests or community activities?

Tell me about the streets, did you spend a lot of time in streets, who did you hangout with? Was it violent?

Institutionalized (from juvenile detention, arrested, foster care, hospital)

Sources of income? (illegal? legal?)

First experience with drugs, any drugs. Tell me about the time you first used drugs…

IF FIRST INJECTION, tell us how it came about, mechanics of injection, syringe source, what type of injection, with whom, where and what? Did you learn anything related to safe injection? And if it was incorporated in your behavior?

Note to interviewer: Keep in mind that first sex and first injection will in some or many cases take place later (maybe much later) than early teens

| EmbeddednessInjection Drug Use | So you were telling me about this “place #1” (specific site where injecting took place) (e.g. John’s house, hallway) where you were injecting with “X, Y and Z” (people with whom injection took place) (e.g. John, a bunch of regulars, most people you didn’t know)  What did you do? (not to inject there, share, not share, share only with partner)  Where the needles came from (source)?  So most people shared… How did you manage no to?  Were people upset that you did not share?  Did they try to dissuade you? How did you explain it to them? |
| --- | --- |
| Note | *Note to interviewer: Norms regarding needle sharing are situational (depend on location and actors present (e.g. subject/co-injectors/other injectors/others) GOALS: Get the Norms of the specific site (e.g. at P’s shooting gallery everybody gets the needle from the box; at M’s apartment you got to bring your own except if you go to the back room-that costs you more though).*  *How did he/she manage to circumvent the norms? Were people upset?* |
| Note 2 | “Embeddedness”. Should include learning safe practices, what others were doing, what pressures they put on you… |

IF First Sexual Experiences, with who, condom use, other homosexual/heterosexual? Did you learn anything related to safe sex? And if it was incorporated in your behavior?

| EmbeddednessSex | So you were telling me about this “partner X” with whom you had sex …  Did you use condoms?  Who had the condoms (source)?  So X did not like condoms … how did you manage using one with him/her?  Was the partner upset because you wanted to use condoms?  How did you explain it to them?  Was he/she upset? Did he/she try to dissuade you? |
| --- | --- |
| *Note:* | Norms regarding condom use) are situational (depend on location and actors present ( subject/partner(s)/others having sex/others present). GOALS: Get the Norms of the actor and specific site if applicable *What were respondents’ actions? (use condoms, no condoms use, suggest condom use) How did he/she manage to circumvent the partners’ norm regarding condom use?* |

| **Staying Safe** | What did you do to stay safe?  How did it change during this period?  What obstacles or support led to this change? |
| --- | --- |
|  |  |

**Period II [Late Teens, 16-19 years old)**

What were the major differences between this time period and the previous time period?

Sources of income. How you were supporting yourself? How did you make money?

Relationships with family. Did you live at your mother’s (father) house? Did you keep in touch with siblings?

Neighborhood life. Who did you hang out? Where did you hang out? Did you hangout in the neighborhood, move around and/or you travel out of neighborhood (Puerto Rico, The South, etc.)

Sexual Experiences. With whom did you have sex? Did you use condoms? Where did you have sex (place)? What types of sex (oral-anal-vaginal)? Did you learn anything related to safe sex with X, Y, Z partner ? How it was incorporated in your behavior (e.g. since that day did you “did that –use condoms, avoid sex with drug users ” most of the times? Long Term partnerships? Children? Abortions?

| EmbeddednessSex | So you were telling me about this “partner X” with whom you had sex …  Did you use condoms?  Who had the condoms (source)?  So X did not like condoms … how did you manage using one with him/her?  Was the partner upset because you wanted to use condoms?  How did you explain it to them?  Was he/she upset? Did he/she try to dissuade you? |
| --- | --- |
| *Note:* | Norms regarding condom use) are situational (depend on location and actors present ( subject/partner(s)/others having sex/others present). GOALS: Get the Norms of the actor and specific site if applicable *What were respondents’ actions? (use condoms, no condoms use, suggest condom use) How did he/she manage to circumvent the partners’ norm regarding condom use?* |

Drug Use. What was your main drug? Did you use any other drugs? Tell about your periods of withdraw, stayed clean, relapse and drug treatments.

INJECTION, with whom? Where? Did you always injected the same way with different people? Did you learn anything related to safe injection? And if it was incorporated in your behavior? What safety practices did you learn from others? How they where incorporated?

| EmbeddednessDrug Use | So you were telling me about this “place #1” (specific site where injecting took place) (e.g. John’s house, hallway) where you were injecting with “X, Y and Z” (people with whom injection took place) (e.g. John, a bunch of regulars, most people you didn’t know)  Did you share?  Where the needles came from (source)?  So most people shared… How did you manage no to?  Were people upset that you did not share?  How did you explain it to them? |
| --- | --- |
|  |  |

Housing and Institutionalized (arrest, jail, prison, hospital, mental, treatment programs…)

Where you tested for HIV? Where? Why? Did someone recommend it? How many times? What was the resulted? Who did you tell?

| **Staying Safe** | What did you do to stay safe?  How did it change during this period?  What obstacles or support led to this change? |
| --- | --- |
|  |  |

**Period III [Early Adulthood 20-30years old)**

What were the differences between this time period and the previous time period?

Sources of income

Relationships with family

Relationships with network

Neighborhood Context, Where were you hanging out? With who?

INJECTION, with whom? Where? Did you always injected the same way with different people? What safety practices did you learn from others?

| EmbeddednessDrug Use | So you were telling me about this “place #1” (specific site where injecting took place) (e.g. John’s house, hallway) where you were injecting with “X, Y and Z” (people with whom injection took place) (e.g. John, a bunch of regulars, most people you didn’t know)  Did you share?  Where the needles came from (source)?  So most people shared… How did you manage no to?  Were people upset that you did not share?  How did you explain it to them? |
| --- | --- |
|  |  |

Housing and Institutionalized (arrest, jail, prison, hospital, mental, treatment programs…)

Where you tested for HIV? Where? Why? Did someone recommend it? How many times? What was the resulted? Who did you tell?

Any other social support?

Tell about your periods of withdraw, stayed clean, relapse and any type of drug treatments.

Sexual Experiences with whom? And condoms, where (place) oral-anal-vaginal. Did you learn anything related to safe sex? And if it was incorporated in your behavior?

Long Term partnerships? Children? Abortions?

| EmbeddednessSex | So you were telling me about this “partner X” with whom you had sex …  Did you use condoms?  Who had the condoms (source)?  So X did not like condoms … how did you manage using one with him/her?  Was the partner upset because you wanted to use condoms?  How did you explain it to them? Was he/she upset? Did he/she try to dissuade you? |
| --- | --- |
|  |  |

| **Staying Safe** | What did you do to stay safe?  How did it change during this period?  What obstacles or support led to this change?  Any other behavior aimed at avoiding risk?  How did you keep vigilant while high?  Any other vigilant behavior? |
| --- | --- |
|  |  |

**Period IV [Late Adulthood 30-40 years old)**

REPEAT until all time periods are covered.

After the time periods respondent will be asked regarding STRATEGIES.

NOTE TO GROUP: THERE IS A CONSENSUS: THIS SECTION NEEDS WORK.

Note to interviewers: The strategy (plan, approach, line of prevention) are guiding principles, the framework under which safe situational responses and other avoidance behaviors are organized - (e.g. not going to buy drugs, keep always a job, reside at mom’s house). The strategy *is* intentional; and its motivation *is* to avoid HIV [and/or HCV ??] (I don’t share needles because I don’t want to get HIV) [[QUESTION: Should other motivations be included?? (e.g. I don’t share needles because I don’t like others blood)]]

Some actions might help prevent HIV but are not be a byproduct of an overall HIV prevention strategy. (e.g. I never went to a shooting gallery because I don’t want to be associated with those low lives)

| Stay safeStrategy - Drug Use | We have talked about the different things you did to avoid HIV while injecting drugs. Was that part of an overall strategy?Did somebody help you to come out with this strategy? How did the strategy change/improve? Did the people that you injected with share this strategy?Do you know of others who have similar strategies? |
| --- | --- |
| Stay safeStrategy - Sex | We have talked about the different things you did to avoid HIV while having sex. Was that part of an overall strategy? How did the strategy change/improve?  Did somebody help you to come out with this strategy?  Do you know of others who have similar strategies? |
